# Influenza C Virus in Cattle with Respiratory Disease, United States, 2016–2018

**DOI:** 10.3201/eid2410.180589

**Published:** 2018-10

**Authors:** Hewei Zhang, Elizabeth Porter, Molly Lohman, Nanyan Lu, Lalitha Peddireddi, Gregg Hanzlicek, Douglas Marthaler, Xuming Liu, Jianfa Bai

**Affiliations:** Chinese Academy of Agricultural Sciences, Institute of Special Economic Animal and Plant Sciences, Changchun, China (H. Zhang);; Kansas State University Veterinary Diagnostic Laboratory, Manhattan, Kansas, USA (H. Zhang, E. Porter, M. Lohman, N. Lu, L. Peddireddi, G. Hanzlicek, D. Marthaler, X. Liu, J. Bai)

**Keywords:** influenza C virus, cattle, bovine respiratory disease, BRDC, bovine respiratory disease complex, United States, USA, Midwest, Oklahoma, Montana, Texas, Kansas, Missouri, Colorado, Nebraska, livestock, cows, zoonoses, respiratory infections, viruses, influenza virus, influenza

## Abstract

We identified influenza C virus (ICV) in samples from US cattle with bovine respiratory disease through real-time PCR testing and sequencing. Bovine ICV isolates had high nucleotide identities (≈98%) with each other and were closely related to human ICV strains (≈95%). Further research is needed to determine bovine ICV’s zoonotic potential.

Influenza viruses are contagious zoonotic pathogens that belong to the *Orthomyxoviridae* family, which consists of 4 genera: *Alphainfluenzavirus* (influenza A virus), *Betainfluenzavirus* (influenza B virus), *Gammainfluenzavirus* (influenza C virus [ICV]), and *Deltainfluenzavirus* (influenza D virus) (*1*–*4*). Classification of influenza viruses is based on the antigenic differences in the nucleoprotein and matrix protein and supported by intergenic homologies of 20%–30% and intragenic homologies >85% (*3*).

The most common influenza pathogen is influenza A virus, which can infect humans, pigs, cattle, birds, as well as other animals (*2*,*4*). ICV was first identified in humans in 1947. This group of influenza viruses was initially thought to exclusively infect humans until isolates were identified in pigs in China (*5*,*6*) and Japan (*7*). Antigenic and genetic analyses suggest that ICV might transmit between humans and pigs in nature (*8*); however, interspecies transmission has not been confirmed experimentally. In 2011, an influenza C–like virus was identified in swine and cattle in the United States (*9*); this virus was initially proposed to be an ICV subtype but was later identified as influenza D virus (*3*) because the virus had ≈50% overall amino acid identity with human ICV strains, a level of divergence similar to that between influenza A and influenza B viruses.

Although influenza viruses of other genera can infect cattle, the potential for ICV infection in cattle has not been previously investigated. The objective of this study was to determine if ICV can be found in specimens from cattle with bovine respiratory disease and, if so, determine the prevalence.

## The Study

Bovine respiratory disease complex (BRDC) is one of the most common causes of death in livestock in US feedlots and feedlots worldwide (*10*). During October 2016–January 2018, we collected 1,525 samples (mainly nasal swab and lung tissue specimens) from cattle in the Midwest of the United States and submitted them to Kansas State Veterinary Diagnostic Laboratory (Manhattan, Kansas, USA) for BRDC diagnostic testing. We screened samples for ICV by real-time reverse transcription PCR, as well as for 10 other BRDC-associated pathogens (*Mannheimia haemolytica*, *Pasteurella multocida*, *Histophilus somni*, *Bibersteinia trehalosi*, *Mycoplasma bovis*, bovine viral diarrhea virus, bovine respiratory syncytial virus, bovine respiratory coronavirus, bovine herpesvirus 1, and influenza D virus; Technical Appendix). We sequenced a 590-bp fragment of the matrix gene from 12 ICV-positive samples (GenBank accession nos. MH421865–73; Technical Appendix Table) to confirm the PCR results and perform a phylogenetic analysis. We selected 1 isolate (C/bovine/Montana/12/2016) for complete genome sequencing (GenBank accession nos. MH348113–9).

Of 1,525 samples, 64 (4.20%) were positive for ICV: 38 samples with a cycle threshold (C_t_) <36 and 26 with a C_t_ 36–39. The most common pathogens were bovine respiratory coronavirus (34.98%), *M. bovis* (32.27%), and *M*. *haemolytica* (17.04%). The remaining BRDC pathogens were present but less prevalent: *P*. *multocida* (13.42%), *H. somni* (12.58%), influenza D virus (11.93%), bovine respiratory syncytial virus (9.19%), bovine viral diarrhea virus (7.05%), *B. trehalosi* (3.47%), and bovine herpesvirus 1 (2.95%).

Co-infections with >1 pathogen are common in BRDC cases. ICV-positive samples were also found to be positive for >1 bovine respiratory disease pathogen (n = 12, Table 1), the most common being *M. bovis* (9/12), followed by *H. somni* (7/12), and *M. haemolytica* (6/12). Among the ICV-positive samples, ICV12 was strongly positive (C_t_ 15.81); this sample was also positive for *M. haemolytica* and *P. multocida*, both bacterial pathogens commonly associated with secondary infections. Other BRDC pathogens associated with secondary infections (*M. bovis*, bovine viral diarrhea virus, and *H. somni*) were also detected in samples ICV4, ICV16, ICV18, and ICV20 (*11*–*13*). These results suggest that ICV is associated with bovine respiratory disease in cattle.

**Table 1:** Cycle thresholds for ICV and other bovine respiratory pathogens in 12 ICV strong positive samples from cattle with respiratory disease, United States, October 2016–January 2018*

ID no.	State	ICV	BVDV	BHV-1	BRSV	BCoV	IDV	*Mycoplasma bovis*	*Mannheimia haemolytica*	*Pasteurella multocida*	*Histophilus somni*	*Bibersteinia trehalosi*
ICV1†	TX	29.95	–	–	–	–	36.77	39.41	–	–	–	–
ICV2†	OK	23.92	–	–	–	–	24.25	29.17	31.30	–	31.32	–
ICV3†	OK	21.02	–	–	38.93	27.00	29.15	31.40	NT	NT	NT	NT
ICV4‡	OK	29.98	–	–	–	–	–	24.54	31.33	–	31.05	–
ICV5†	MO	24.47	–	34.98	–	–	–	30.12	–	28.00	24.40	34.00
ICV6†	CO	26.91	–	–	–	–	–	30.25	32.78	29.60	30.00	35.00
ICV12†	MT	15.81	–	–	–	–	–	–	22.87	25.70	–	–
ICV16‡	NE	27.18	16.44	–	–	–	–	28.47	–	–	–	–
ICV18†	MN	30.58	–	–	–	–	–	–	35.06	–	–	–
ICV20‡	KS	27.92	–	–	–	–	–	–	–	–	23.49	–
ICV21‡	KS	26.72	–	–	35.59	–	–	25.67	30.95	–	28.47	–
ICV22†	MT	25.08	–	–	–	–	20.24	35.58	–	–	35.64	–

*BCoV, bovine respiratory corona virus; BHV-1, bovine herpesvirus 1; BRSV, bovine respiratory syncytial virus; BVDV, bovine viral diarrhea virus; ICV, influenza C virus; ID, identification; IDV, influenza D virus; NT, not tested (sample used up); –, negative. †Nasal swab sample used in analysis. ‡Lung sample used in analysis.

We further evaluated 12 strong positive (C_t_<31) samples by sequencing a 590-bp fragment of their matrix gene. Alignment of the partial matrix gene sequences indicated that the isolates in 3 samples (ICV2, ICV3, and ICV4) obtained from different cattle on the same farm in Oklahoma were identical. Because these 3 influenza viruses were most likely the same strain, the virus in just 1 sample (ICV2) was used for phylogenetic analysis. The matrix gene sequence in sample ICV5 from Missouri (GenBank accession no. MH421866) was identical to that in ICV6 from Colorado (GenBank accession no. MH421867).

Phylogenetic analysis indicated that the bovine ICV isolates are closely related to the porcine and human ICV isolates, and the bovine ICV isolates are more closely related to each other (Table 2; Figure). The bovine ICV isolates’ partial matrix gene sequences shared high nucleotide identities (≈98%). For both partial matrix gene sequences and the whole genome sequence (7 segments), the nucleotide identity between bovine and human isolates was ≈95%. The full genome sequence of C/bovine/Montana/12/2016 from sample ICV12 had high nucleotide identity to C/Mississippi/80 (and several other human ICV strains), with an overall identity of 97.1%. Nucleotide identities between these 2 isolates were also high for each gene: 97.0% for polymerase basic 2, 97.7% for polymerase basic 1, 97.5% for polymerase 3, 96.2% for hemagglutininesterase, 96.8% for nucleoprotein, 96.8% for matrix, and 97.6% for nonstructural protein. The only porcine ICV isolate available was more closely related to human (≈98% identity) isolates than bovine (≈95% identity) isolates; the porcine ICV isolate had nearly the same identity that the human ICV isolates had among each other (Table 2).

**Table 2:** Average nucleotide identities among bovine, porcine, and human ICV strains, United States*

Gene sequence	Bovine ICV, %	Human ICV, %	Bovine ICV vs. human ICV, %	Bovine ICV vs. porcine ICV, %	Human ICV vs. porcine ICV, %
Matrix, partial	98.43	98.47	95.54	96.06	98.84
Polymerase basic 2	NA	97.76	94.97	95.00	98.34
Polymerase basic 1, full length	NA	97.59	94.69	94.70	98.08
Polymerase 3	NA	97.79	96.36	95.50	97.31
Hemagglutinin esterase	NA	95.44	90.83	91.10	95.97
Nucleoprotein	NA	97.67	95.48	95.30	97.85
Matrix, complete	NA	98.33	95.51	95.80	98.82
Nonstructural protein	NA	98.32	95.67	95.80	98.36
Entire genome		97.56	94.79	94.74	97.82

*ICV, influenza C virus; NA, not applicable.

**Figure:**
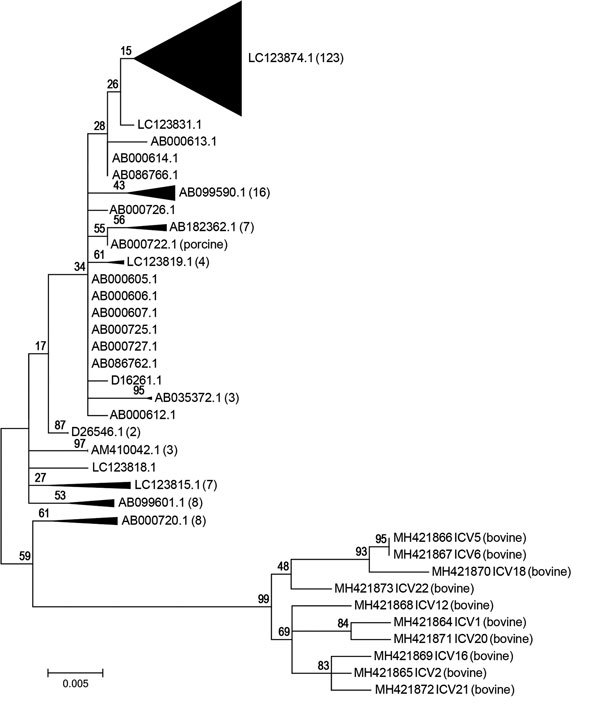
Phylogenetic tree of 10 bovine ICV isolates compared with 1 porcine and 195 human ICV isolates (not labeled), United States. Some subtrees containing only human isolates were collapsed to decrease the size of the image. A representative taxon identification number for each collapsed subtree is shown with the number of taxa in the subtree in parentheses. The percentage of trees in which the associated taxa clustered together is shown above the branches. The tree with the highest log likelihood is shown. The tree is drawn to scale, with branch lengths in the same units as those of the evolutionary distances used to infer the phylogenetic tree. Scale bar indicates the relative distance of 0.005, which indicates a 0.5% sequence difference. ICV, influenza C virus.

The phylogenetic tree of the partial matrix gene sequences (Figure) further demonstrates the relationship between bovine and human ICV isolates. All bovine ICVs formed a separate clade on the phylogenetic tree, with a 99% bootstrap value. Of the 195 partial matrix gene sequences from human ICVs, the 10 corresponding sequences from bovine ICVs had the highest identities (average 96.70%) to those from C/Mississippi/80 (GenBank no. AB000720.1), C/Nara/82 (GenBank no. AB000723), and C/Kyoto/41/82 (GenBank no. AB000724) and the lowest identities (average 94.21%) to those from C/Yamagata/30/2014 (GenBank no. LC123874) and C/Yamagata/32/2014 (GenBank no. LC123875).

## Conclusions

This study confirms the presence of ICV in US cattle with clinical signs of bovine respiratory disease. Although interspecies transmission of influenza viruses occurs between humans and other animals, we do not have data that indicates ICV is a zoonotic pathogen. However, the full genome sequence of C/bovine/Montana/12/2016 has 97.1% nucleotide identity with the human isolate C/Mississippi/80, which is within the range of average identities among human isolates. More detailed investigations are needed to confirm if ICV is involved in bovine respiratory disease, to characterize the relationship between bovine and human ICV strains, and to determine the zoonotic potential of bovine ICV isolates to cause human disease.

Technical Appendix Description of methods used for PCR and phylogenetic analysis and bovine influenza C virus sequence information.
