# Supplementary material for: Influenza C Virus in Cattle with Respiratory Disease, United States, 2016–2018
Source: Emerg Infect Dis. 2018 Oct;24(10):1926–9. doi: 10.3201/eid2410.180589 (PMC6154146; doi:10.3201/eid2410.180589)
Supplement: Technical Appendix — Description of methods used for PCR and phylogenetic analysis and bovine influenza C virus sequence information. [file 18-0589-Techapp-s1.pdf]

Article DOI: https://doi.org/10.3201/eid2410.180589

Influenza C Virus in Cattle with Respiratory Disease, United States, 2016–2018

Technical Appendix

Real-Time Reverse Transcription PCR

Bovine clinical samples were homogenized, and the nucleic acids were extracted by using QIAamp Viral RNA Mini Kit (QIAGEN, Valencia, CA, USA). Two sets of nonoverlapping real-time reverse transcription (RT) PCR primers and probes (labeled with the same dye) were designed to target the influenza C virus (ICV) matrix gene. The primers and probe of set 1 (amplicon size 144 bp): ICV1-F (5′-TCGGCAGATGGGAGAGATG-3′), ICV1-R (5′-GAATTGGTGAGTTGTCGGTTTC-3′), and ICV1-Pr (MAX-5′-CTCCCAGGTCAAGTCTCTCCCT-3′-IBFQ), and the primers and probe of set 2 (amplicon size 100 bp): ICV2-F (5′-TGGCCTTGGAGAAGAAGCA-3′), ICV2-R (5′-CAAGTGGGGTCTCATTATATTACTTCC-3′), and ICV2-Pr (MAX-3′-TGATTRCATAATATGGCCAAACTTTCTG-5′-IBFQ) were synthesized from Integrated DNA Technologies, Inc. (Coralville, IA, USA). In silico analysis indicated that the 2 sets of assays covered 195 of 196 bp (99.5%) of the complete and near-complete ICV matrix gene sequences available. We performed real-time reverse RT-PCR tests using a 20-µL reaction volume (0.4 µM forward and reverse primers, 0.2 µM probe, 3 µL template nucleic acid, 2 µL 10× Multiplex Enzyme Mix, and 10 µL 2× Multiplex RT-PCR Buffer) and the Path-ID Multiplex One-Step RT-PCR Kit (Applied Biosystems, Grand Island, NY, USA). The thermocycling parameters included a RT step at 48°C for 10 min and RT inactivation and denaturation at 95°C for 10 min, followed by 45 cycles of denaturation at 95°C for 15 sec and annealing and extension at 60°C for 45 sec. The cycle threshold was determined with the CFX96 Touch Real-Time PCR Detection System (Bio-Rad, Hercules, CA, USA) and analyzed by using Bio-Rad CFX Manager 3 software.

Partial Matrix Gene Sequencing of ICV-Positive Samples and Complete Genome Sequencing of Sample ICV12

A pair of sequencing primers flanking the real-time PCR target were designed to amplify a 590-bp fragment of ICV matrix gene for Sanger sequencing confirmation of PCR-positive samples. The primer sequences were ICV-cF (5′-AAAGCCAGCACAGCAATGAA-3′) and ICV-cR (5′-TCAAAAATACCATCATTGGAAAAAGG-3′). The ICV matrix gene fragment was amplified by using LA Taq PCR Kit (TaKaRa, Mountain View, CA, USA) from cDNA synthesized with the SuperScript III First-Strand Synthesis Supermix Kit (Invitrogen, Carlsbad, CA, USA). PCR products were sequenced by an outsourced sequencing facility (Genewiz, South Plainfield, NJ, USA). Sample ICV12 was selected for complete genome sequencing as previously described (*1*,*2*).

Phylogenetic Analyses

A total of 195 complete and near-complete human ICV matrix segment sequences and 1 porcine ICV matrix segment sequence were obtained from GenBank (same as the sequences from Influenza Research Database). The analyses of 10 bovine and human ICV matrix gene sequences were carried out by using CLC Genomic Workbench version 9.0.1 (CLC bio, Boston, MA, USA) to generate a multiple sequence alignment. BioEdit version 7.2.5 (https://www.bioedit.com/) was used to generate a sequence identity table.

We constructed a phylogenetic tree with MEGA7 (*3*) using multiple alignments of the 10 bovine ICV matrix gene sequences generated in this study plus 195 complete and near-complete human ICV matrix gene sequences and 1 porcine ICV matrix gene sequence. The alignment length was trimmed to 559 bp. The evolutionary history was inferred by using the maximum likelihood method on the basis of the Jukes-Cantor model (*4*) with 1,000 bootstrap replicates (*5*). Initial trees for the heuristic search were obtained automatically by applying the Neighbor-Join and BioNJ algorithms to a matrix of pairwise distances estimated by using the maximum composite likelihood approach and then selecting the topology with the superior log likelihood value.

References

1. Roy Mukherjee T, Mukherjee A, Mullick S, Chawla-Sarkar M. Full genome analysis and characterization of influenza C virus identified in eastern India. Infect Genet Evol. 2013;16:419–25. PubMed http://dx.doi.org/10.1016/j.meegid.2013.02.014

2. Matsuzaki Y, Sugawara K, Furuse Y, Shimotai Y, Hongo S, Oshitani H, et al. Genetic lineage and reassortment of influenza C viruses circulating between 1947 and 2014. J Virol. 2016;90:8251–65. PubMed http://dx.doi.org/10.1128/JVI.00969-16

3. Kumar S, Stecher G, Tamura K. MEGA7: Molecular Evolutionary Genetics Analysis version 7.0 for bigger datasets. Mol Biol Evol. 2016;33:1870–4. PubMed http://dx.doi.org/10.1093/molbev/msw054

4. Jukes TH, Cantor CR. Evolution of protein molecules. In: Munro HN, editor. Mammalian protein metabolism. New York: Academic Press; 1969. pp. 21–132.

5. Felsenstein J. Confidence limits on phylogenies: an approach using the bootstrap. Evolution. 1985;39:783–91. PubMed http://dx.doi.org/10.1111/j.1558-5646.1985.tb00420.x

Technical Appendix Table. Sample and sequence information of bovine ICV isolates acquired from cattle with respiratory disease, United States, October 2016–January 2018*

Sample ID	Sample source	Bovine ICV isolate	GenBank accession no.
ICV1	Nasal swab	C/bovine/Texas/1/2016	MH421864
ICV2	Nasal swab	C/bovine/Oklahoma/2/2017	MH421865
ICV3	Nasal swab	C/bovine/Oklahoma/3/2017	NA
ICV4	Lung	C/bovine/Oklahoma/4/2017	NA
ICV5	Nasal swab	C/bovine/Missouri/5/2017	MH421866
ICV6	Nasal swab	C/bovine/Colorado/6/2017	MH421867
ICV12	Nasal swab	C/bovine/Montana/12/2016	MH421868†
ICV16	Lung	C/bovine/Nebraska/16/2017	MH421869
ICV18	Nasal swab	C/bovine/Minnesota/18/2017	MH421870
ICV20	Lung	C/bovine/Kansas/20/2017	MH421871
ICV21	Lung	C/bovine/Kansas/21/2017	MH421872
ICV22	Nasal swab	C/bovine/Montana/22/2016	MH421873

*ICV, influenza C virus; ID, identification; NA, not applicable.
†GenBank accession nos. for complete genome are MH348113–MH348119.
